# Insuficiência Cardíaca por Doença de Chagas: Desafio ainda a Ser Vencido no Século 21

**DOI:** 10.36660/abc.20250332

**Published:** 2025-06-26

**Authors:** Roberto M. Saraiva, Luciana F. Portela, Andréa R. Costa

**Affiliations:** 1 Fundação Oswaldo Cruz Instituto Nacional de Infectologia Evandro Chagas Rio de Janeiro RJ Brasil Instituto Nacional de Infectologia Evandro Chagas, Fundação Oswaldo Cruz, Rio de Janeiro, RJ – Brasil

**Keywords:** Doença de Chagas, Insuficiência Cardíaca, Prognóstico

Embora os novos casos de doença de Chagas (DC) tenham diminuído nas últimas décadas por meio do controle da transmissão vetorial e transfusional, cerca de 3,7 milhões de brasileiros ainda vivem cronicamente afetados por essa doença.^1^ Como cerca de 30 a 40% dessa população apresenta a forma cardíaca (CCC), e a insuficiência cardíaca (IC) está presente em cerca de 10% dos portadores de CCC,^2^ a DC é uma das principais causas de IC em países endêmicos, variando de 4% a 50%.^2-4^ O artigo de Silva et al. acrescenta importantes informações epidemiológicas atualizadas: 8,7% dos pacientes admitidos por IC aguda em um grande registro multicêntrico brasileiro entre 2011 e 2018 apresentavam CCC.^5^ Portanto, mesmo atualmente, ainda é imperativo incluir DC na investigação diagnóstica de pacientes com IC de etiologia desconhecida. Outro aspecto importante da IC por DC é que ela apresenta maior mortalidade^4,6,7^ e incidência de AVC^4^ do que a IC relacionada a outras etiologias. O artigo de Silva et al. confirmou que, entre aqueles admitidos por IC aguda, os pacientes com DC apresentaram uma maior taxa de morte ou transplante cardíaco durante a internação hospitalar e maior incidência cumulativa de morte após 3, 6 e 12 meses após a alta.^5^

O pior desfecho na DC pode estar relacionado a características específicas da doença, incluindo miocardite fibrosante progressiva com aumento cumulativo da fibrose miocárdica ao longo do tempo,^8^ fenômenos cardioembólicos mais frequentes,^4^ arritmias com risco de vida mais frequentes e pior hemodinâmica e função sistólica do ventrículo esquerdo (VE).^9^ De fato, Silva et al. descreveram uma menor pressão arterial sistólica e fração de ejeção do VE entre pacientes com DC.^5^ Outro aspecto descrito por Silva et al. é que sinais de IC direita foram mais frequentes em pacientes com CCC.^5^

Apesar do pior prognóstico da IC relacionada a DC, faltam estudos específicos sobre a previsão de um novo caso de IC ou sobre o tratamento específico da IC em pacientes com DC. Um estudo recente revelou que a maioria dos preditores tradicionais de novos casos de IC não foram associados a novos casos em pacientes com CCC. Ao mesmo tempo, parâmetros ecocardiográficos, incluindo função sistólica e diastólica do VE e parâmetros derivados do strain, podem ser bons preditores de novos casos de IC em CCC.^10^ Portanto, muitos desafios ainda precisam ser superados para reverter o excesso de mortalidade relacionado à CCC. Esses desafios incluem o baixo financiamento público e privado para ensaios clínicos multicêntricos para investigar novos medicamentos, dispositivos cardíacos e abordagens não farmacológicas para CCC e para avaliar novos tratamentos antiparasitários que poderiam melhorar a progressão da DC, ausência de treinamento contínuo para profissionais de saúde e políticas públicas insuficientes visando diagnóstico e tratamento precoces, entre outros.^11^
