# Supplementary material for: Insuficiência Cardíaca por Doença de Chagas: Desafio ainda a Ser Vencido no Século 21
Source: Arq Bras Cardiol. 2025 Jun 26;122(5):e20250332. [Article in Portuguese] doi: 10.36660/abc.20250332 (PMC12269903; doi:10.36660/abc.20250332)
Supplement: Translation

# Translation: Acute Heart Failure Due to Chagas Disease: Still a Challenge in the 21^st^ Century

**DOI:** 10.36660/abc.20250332i

**Authors:** Roberto M. Saraiva, Luciana F. Portela, Andréa R. Costa

**Affiliations:** 1 Rio de Janeiro RJ Brazil Instituto Nacional de Infectologia Evandro Chagas, Fundação Oswaldo Cruz, Rio de Janeiro, RJ – Brazil

**Keywords:** Chagas Disease, Heart Failure, Prognosis

Although new Chagas disease (CD) cases have decreased in the last decades through control of vector-borne and blood transfusion transmission, around 3,7 million Brazilians still live chronically affected by this disease.^1^ As around 30-40% of this population presents the cardiac form (CCC), and heart failure (HF) is present in about 10% of those with CCC,^2^ CD is a major cause of HF in endemic countries, ranging from 4% to 50%.^2-4^ The paper by Silva et al. adds important updated epidemiological information that 8.7% of the patients admitted due to acute HF in a large Brazilian multicenter registry between 2011 and 2018 had CCC.^5^ Therefore, even nowadays, it is still imperative to include CD in the diagnosis workup of patients with HF of unknown etiology. Another important aspect of the HF due to CD is that it presents higher mortality^4,6,7^ and stroke incidence^4^ than HF related to other etiologies. The paper by Silva et al confirmed that among those admitted due to acute HF, patients with CD had a higher rate of the composite of death or heart transplant during hospital stay and a higher cumulative incidence of death after 3, 6, and 12 months after discharge.^5^

The worst outcome in CD may be related to specific characteristics of the disease, including a progressive fibrosing myocarditis with a cumulative increase in myocardial fibrosis over time,^8^ more frequent cardioembolic phenomena,^4^ more frequent life-threatening arrhythmias, and worse hemodynamics and left ventricular (LV) systolic function.^9^ In fact, Silva et al. described a lower systolic blood pressure and LV ejection fraction among patients with CD.^5^ Another aspect described by Silva et al is that signs of right-sided HF were more frequent in patients with CCC.^5^

Although the worst outcome, specific studies on predicting incident HF or addressing HF treatment specifically in patients with CD are lacking. A recent study revealed that most traditional incident HF predictors were not associated with incident HF in patients with CCC. At the same time, echocardiographic parameters, including LV systolic and diastolic function and strain-derived parameters, can be good predictors of incident HF in CCC.^10^ Therefore, many challenges are still to be overcome in order to reverse the excess in mortality related to CCC. Those challenges include low public and private funding for multicenter clinical trials to investigate new drugs, cardiac devices, and non-pharmacological approaches for CCC and to evaluate new anti-parasitic treatments that could ameliorate CD progression, absence of continuous training for health-care professionals, and insufficient public policies targeting early diagnosis and treatment, among others.^11^
